# Hsp90 Blockers Inhibit Adipocyte Differentiation and Fat Mass Accumulation

**DOI:** 10.1371/journal.pone.0094127

**Published:** 2014-04-04

**Authors:** Sébastien Desarzens, Wan-Hui Liao, Caterina Mammi, Massimiliano Caprio, Nourdine Faresse

**Affiliations:** 1 University of Zurich, Zurich, Switzerland; 2 IRCCS San Raffaele, Roma, Italy; University of Geneva, Switzerland

## Abstract

Geldanamycin derivatives are benzoquinone ansamycin antibiotics that bind to Hsp90 and alter its function. The alteration of Hsp90 activity limits some cellular hormonal responses by inhibiting nuclear receptors activation. The nuclear receptors activity, such as PPARγ, the mineralocorticoid and glucocorticoid receptors (MR and GR) play a critical role in the conversion of preadipocytes to mature adipocytes. Given the importance of these nuclear receptors for adipogenesis, we investigated the effects of geldanamycin analogues (GA) on adipocyte differentiation and function. We found that early exposure of preadipocyte cells to GA inhibited their conversion into mature adipocytes by inhibiting the adipogenic transcriptional program and lipid droplets accumulation. Furthermore, GA altered the adipokines secretion profile of mature adipocyte. The anti-adipogenic effect of GA was also confirmed in mice fed a high fat diet. Biochemical analysis revealed that anti-adipogenic effects of geldanamycin analogues may result from the simultaneous inhibition of MR, GR and PPARγ activity. Taken together, our observations lead us to propose Hsp90 as a potent target for drug development in the control of obesity and its related metabolic complications.

## Introduction

Adipogenesis represents the complex cascade of events leading a preadipocyte to acquire the feature of a mature adipocyte. It occurs as a consequence of normal cell turnover, and contribute to adipose tissue expansion in response to hormonal cues and calorie surplus [1]. Excess adipocyte size or number leads to obesity, which is a hallmark of metabolic syndrome (MetS) that includes hypertension, diabetes and dyslipidemia [2]. Obesity affects around 300 million individuals worldwide, a number that is expected to grow continuously in the next years, making obesity and MetS a priority in health expenses [3].

Several hormones and growth factors induce adipogenesis through a tightly controlled transcriptional cascade involving the sequential activation of CCAAT/enhancer binding proteins (C/EBPs) and peroxisome proliferator-activated receptor γ (PPARγ) [4]. Briefly, C/EBPβ and δ induce the expression of PPARγ which is responsible for inducing C/EBPα. Once initiated, this cascade will maintain the expression of these critical transcription factors thanks to a positive feedback loop where C/EBPα and PPARγ reciprocally reinforce their expression [4].

The mineralocorticoid (MR) and glucocorticoid receptors (GR) are expressed in adipocytes and are both involved in adipogenesis. Given the lack of 11βHSD2 in adipocytes, these two receptors can be activated by glucocorticoids [5]. In their non-activated state, these receptors are predominantly cytoplasmic and part of a large heteromeric complex interacting with a number of proteins. Among these, the chaperone protein Heat Shock Protein 90 (Hsp90) is the best characterized. Chaperone proteins play an important role in the conversion of misfolded proteins to a functional conformation. In the case of MR/GR, their association with Hsp90 is crucial for proper ligand binding and receptor function. Indeed, it was shown that disruption of this interaction by geldanamycin, a benzoquinone ansamycin antibiotic, leads to decreased MR and GR mediated transcription [6], [7], [8]. Upon ligand binding, these interactions are disrupted and the cytoplasmic complex is dissociated allowing the translocation of MR/GR into the nucleus to regulate transcription of target genes.

GR is critical for the early adipogenesis [9], but serves a relatively minor role in terminal differentiation. *In vitro* studies showed that knock-down of MR and not GR in 3T3-L1 cells affects the differentiation induced both by mineralocorticoids and glucocorticoids [10], [11]. Contradictory findings were observed in primary human preadipocytes where suppression of GR but not MR blocked glucocorticoids mediated adipogenesis [12]. In addition, studies in animal models of obesity (*ob/ob* and *db/db* mice) showed an increase in MR expression levels in adipose tissue and a reversion of adipocyte dysfunction after treatment with MR blockers [13], [14]. These observations make MR and GR interesting targets for limiting adipose tissue expansion and consequently the appearance of MetS. Interestingly, it was found that MR blockers spironolactone or eplerenone attenuate obesity-related insulin resistance and inflammation [13], [14]. Recently, drospirenone, a powerful anti-mineralocorticoid with progestogenic activity, has been shown to exert an antiadipogenic effect related to an alteration of MR activation [11].

Geldanamycin and its analogues (17-AAG and 17-DMAG) block Hsp90 activity and consequently inhibit nuclear receptors activation by decreasing their expression levels [7], [8]. Numerous preclinical data and multiple phase I and II trials reported safety and promising activity of geldanamycin analogues (GA) in cancer treatment [15], [16], [17], [18], [19]. Given the pharmacological profile of GA and the adipogenic role of MR, GR and PPARγ, we hypothesized that GA prevent adipogenesis and adipocyte function. Very recently, two concomitant *in vitro* studies described the anti-adipogenic effects of Hsp90 blockers on 3T3-L1 cells [20], [21]. In the study of He *et al*., the authors showed that Radicicol, a Hsp90 blocker, inhibited 3T3-L1 differentiation through affecting the PDK1/Akt pathway, leading to the inhibition of mitotic clonal expansion [20]. In the study of Nguyen *et al*., the authors showed that Hsp90 blockers (geldanamycin and novobiocin), inhibit adipocyte differentiation by inducing the destabilization of PPARγ at the protein level [21]. In this study, we found that GA inhibits the adipocyte differentiation and function *in vitro* and fat mass accumulation *in vivo*. This inhibition could be the result of a decrease in GR and PPARγ expression levels and an inhibition of MR activation. Taken together, the beneficial effects of Hsp90 inhibitors in fat mass accumulation provide a new direction for MetS treatment.

## Material and Methods

### Cell culture

Murine 3T3-L1 and 3T3-F442A preadipocytes were cultured in standard medium containing DMEM and 10% FCS. At confluence, the differentiation process of 3T3-F442A cells was initiated using DMEM 10% FCS supplemented with 10 μg/ml insulin for 2 days, and then grown in standard medium for additional 6 days. For 3T3-L1 cells, the differentiation was initiated at confluence by stimulation with 500 μM 3-isobutyl-1-methylxanthine, 10 μg/ml insulin and 1 μM dexamethasone for 2 days. Cells were then grown in DMEM 10% FCS supplemented with 10 μg/ml insulin for additional 8 days. 17-AAG treatment at indicated dose was started from the beginning of the differentiation process. For primary cells culture, gonadal fat was extracted and digested with collagenase type I (1 mg/ml) for 30 min. The totality of the digestion mixture was seeded in an upside down T 25 cm^2^ flasks completely filled with DMEM and 20% FCS in presence or absence of 17-AAG. After 4 days allowing the cells attachment, the flasks were putted back in normal position and cells were grown using standard culture conditions in 37°C 5% CO_2_ for 10 days.

### Oil red-O staining

For determination of lipid content, differentiated cells were fixed in 4% paraformaldehyde in PBS, washed twice with water and stained with oil red-O. After oil red-O removal and washes once with 60% isopropanol and twice with water, cells were visualized under light microscopy. To quantify the lipid content, the stained cells were permeabilized with 10% SDS and the eluate was measured using spectrophotometer at 520 nm.

### Cell cycle and viability

For flow cytometric determination, cells were trypsinized and fixed in 70% ethanol. Fixed cells were stained with Propidium Iodide, and analyzed on a FACScan flow cytometer. For the cell viability the medium and attached cells were collected and death cell were visualized using the trypan blue dye exclusion method.

### Lipolysis assay

Differentiated 3T3-L1 adipocytes were cultured for 48 h with or without 17-AAG (100 nM) in serum free medium and then treated or not with 1 μM isoproterenol for 2 h. Medium was collected and the glycerol released was measured by a colorimetric method using a Free glycerol assay kit (Cell Biolabs).

### Glucose uptake

Differentiated adipocytes were cultured for 48 h with or without 17-AAG (100 nM). Cells were washed with Krebs-Ringer buffer containing 12 mM HEPES (KRH) and then treated with insulin (100 nM) for 20 min. Glucose uptake was initiated by addition of 1 μCi/well of [^3^H]-Deoxy-D-glucose for 10 min and stopped by washing cells 3 times with ice-cold KRH. Cells were lysed with 0.1 M NaOH and radioactivity was measured by scintillation counting. In parallel, the protein concentration of the different wells was evaluated by Bradford method.

### Real-time quantitative PCR

Total RNA of adipocyte was extracted using the NucleoSpin RNA (Macherey-Nagel) and was reverse transcribed using RevertAidTM cDNA Synthesis Kit (Thermo Scientific). Sequences of primers are indicated in the Table S1. Quantitative PCR was performed in replicate for each sample in a Light Cycler 480 Real-Time PCR System by using Master I SYBR Green qPCR Master Mix (Roche) according to the manufacturer's instructions. Relative amounts of mRNA were determined using the Comparative CT Method for quantification and were normalized to GAPDH mRNA expression.

### Immunoblotting and adipokines array

Cells and tissue extracts were prepared as previously described and 30 μg of protein extracts were loaded for SDS-PAGE [22]. The antibodies used were: anti-MR (1/100; kindly provided by Gomez-Sanchez CE), anti-GR (1/1000 Santa Cruz), anti-SGK1 (1/1000 Sigma), anti-HSP90 (1/1000 Cell Signaling), anti-PPARγ (1/500 Cell Signaling), anti-CEBPα (1/500 Cell Signaling) and anti-actin (1/1000 Sigma).

For the adipokine array, cell supernatants were assessed following manufacturer's instructions (Mouse Adipokines Array Kit, R&D System). Briefly, supernatants from 6 independent experiments were collected and pooled in 2 aliquots (pre-adipocytes and mature adipocytes). 300 μl of the collected medium was incubated over-night with the adipokine antibody cocktail and the blocked nitrocellulose membrane. After several washes, detection was performed using a chemi-luminescence kit and autoradiography films. Quantification of the immunoblots was performed using ImageJ software (NIH).

### Animal experiments

Animal studies were carried out in accordance with Swiss animal welfare regulations and after evaluation and written consent of the veterinarian office of the Canton of Vaud, Switzerland (license number 2591.0).Sixteen adult male C57BL/6 mice were divided into 2 groups, one group as vehicle control and the other one treated with 17-DMAG (LC Laboratories).During the experiment, mice were fed with high fat diet (60% of calories derived from fat, Research Diet) and received daily intraperitoneal injection with PBS or 17-DMAG 10 mg/kg of body weight dissolved in PBS. The mice body weight was assessed every 3 days. After 33 days, the body composition was analyzed on individual mouse under light gaz anesthesia (isoflurane), by quantitative nuclear magnetic resonance using an EchoMRI Analyzer (EchoMedical Systems, Houston, TX). Data for individual mice were obtained by averaging results from two consecutive measurements. Then, mice were placed in metabolic cages to measure body weight, water/food consumption and urine collection for 3 days. Urinary Na^+^ and K^+^ were measured using a flame photometer (Cole-Palmer Instruments, Vernon Hills, IL).Mice were then anesthetized by isoflurane inhalation for blood collection and euthanized by cervical dislocation for tissue collection. Plasma glucose, triglycerides, cholesterol were measured by the Clinical Chemistry Lab of the University Hospital in Lausanne using standard techniques.

### Histological analysis

The epididymal and inguinal adipose tissue was fixed with 4% paraformaldehyde in PBS and embedded in paraffin blocs. 10 μm sections were stained with hematoxylin and eosin. Images were acquired under light microscope and the size of adipocytes was determined using ImageJ software (NIH).

### Data analysis

All the experiments are done at least twice, and the representative immunoblots were shown. For all histograms the data are shown as the average ± SD of results of at least three independent experiments. Differences between test and control conditions were assessed by Student's t test analysis or Wilcoxon signed rank test.

## Results

### Hsp90 blockade inhibits lipids accumulation in adipocytes cells in vitro

**Figure 1:**
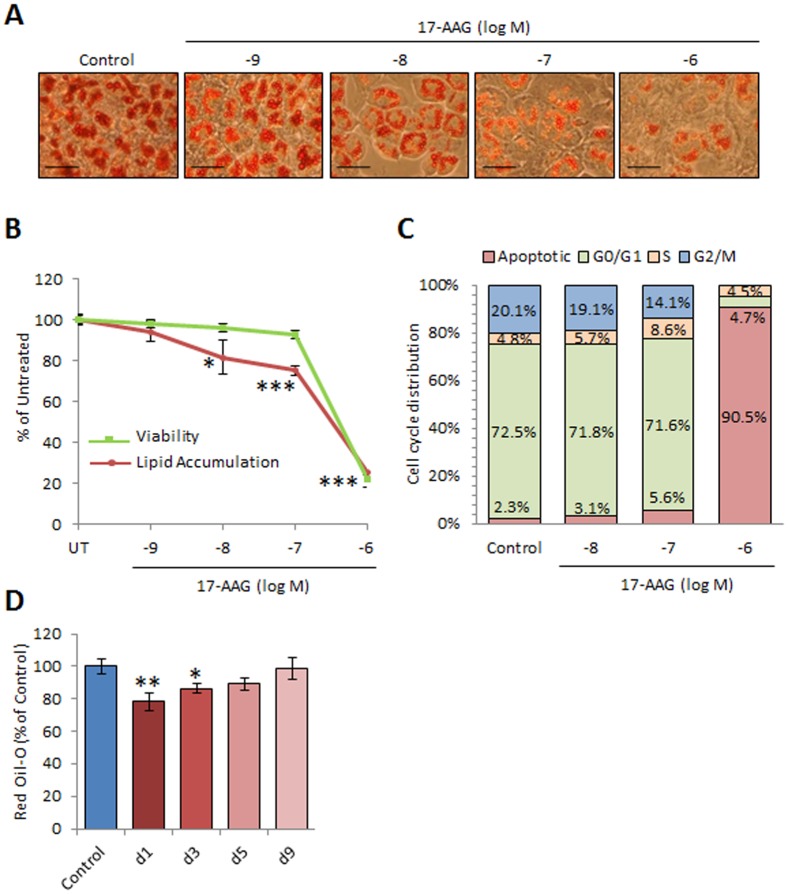
Geldanamycin analogues inhibit lipid accumulation in adipocytes. (A) At confluence, 3T3-L1 preadipocytes were induced to differentiation in presence of an increasing dose of 17-AAG for 9 days. Lipid accumulation was visualized under microscope after Oil-Red-O staining (scale bar, 70 μm) and (B) quantified after extraction of the stained lipid and the absorbance measured at 520 nm. The cell viability was assessed by Trypan blue staining. Data are given as mean ± SD (n = 3), *p<0.05, ***p<0.001respect to untreated (C) Differentiating 3T3-L1 cells were cultured for 3 days with or without 17-AAG (100 nM). Cell cycle was determined on FACScan. Given data are representative of 2 independent experiments performed in triplicate. (D) 3T3-L1 preadipocytes were induced to differentiation and treated with 17-AAG (100 nM) at the indicated day. At day 10, lipid accumulation was stained with Oil-Red-O and quantified. Data are given as mean ± SD (n = 3), *p<0.05, ***p<0.001

To investigate the potential anti-adipogenic effect of Hsp90 blockade, preadipocyte 3T3-L1 cells were differentiated into mature adipocytes in the presence of an increasing dose (from 10^−9^ to 10^−6^ M) of the DMSO soluble geldanamycin analogue17-AAG. Treatment of adipocytes at the beginning of the differentiation process with 17-AAG inhibited lipid accumulation assessed by oil red-O staining in a dose dependent manner (Figure 1A and B). Similar results were obtained using the water soluble geldanamycin analogue 17-DMAG (Figure S1A and B).

To assess the cytotoxic effects of 17-AAG on preadipocytes, we first evaluated the cellular viability by trypan blue assay. We found that concentrations higher than 500 nM induced more than 50% of cell death (Figure 1B). The evaluation of the cellular cycle by FACS analysis showed that the apoptotic fraction dramatically increases with high concentration of 17-AAG (1 μM). Additionally, 17-AAG added in the first days of differentiation inhibited the clonal expansion (G2/M phase) initiated by the differentiation medium (Figure 1C). Due to its significant inhibition on lipid accumulation and the minimal toxicity, the dose of 100 nM was used for all the subsequent *in vitro* experiments.

Finally, to determine the critical time frame for the adipogenesis inhibition by 17-AAG, we treated differentiating adipocytes at different time points. We found that 17-AAG treatments after the day 2 post-induction have no effect on the cellular lipid accumulation (Figure 1D).

### The adipogenic transcriptional program is repressed by Hsp90 blockers

**Figure 2:**
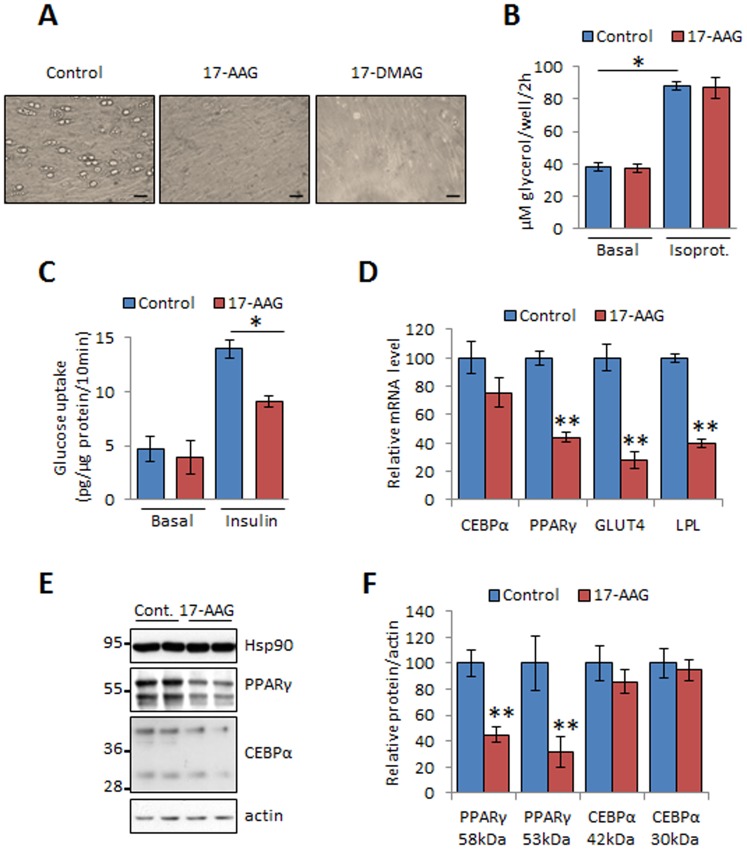
Hsp90 inhibition decreases the adipogenic transcriptional program without any effect on cytotoxicity. (A) Primary preadipocytes derived from white adipose tissue (WAT) of mouse gonadal fat were cultured for 10 days with 17-AAG (300 nM) or DMSO (Control) and visualized by microscopy 40X (scale bar: 50 μm). (B) Mature 3T3-L1 were cultured for 2 days with or without 17-AAG (100 nM) and tested for an additional 2 h for lipolysis in response to 1 μM of isoproterenol. (C) 3T3-L1 cells were induced to differentiation in presence of 17-AAG for 2 days then tested for [^3^H]-Deoxy-D-glucose uptake for 10 min. (D) 3T3-L1 preadipocytes were induced to differentiation in presence or absence of 17-AAG for 10 days. The abundance of CEBPα, PPARγ, LPL and Glut4 mRNA in 3T3-L1 cells was measured by quantitative RT-PCR. Given are means relative to GAPDH of 3 experiments performed in duplicates ± SEM, **p<0.01. (E) Cell lysates were analyzed by immunoblotting using antibodies against Hsp90, PPARγ, CEBPα and actin as a loading control. (F) The protein expression levels were quantified and values are given as mean ± SEM (n = 6), **p<0.01.

The relevance of our findings on 3T3-L1 cells were confirmed in the 3T3-F442A cells, a cell line able to differentiate into fat pads *in vivo* [23], and in primary culture of mouse preadipocytes (Figure S1A and B). Chronic exposure of primary cells derived from epididymal fat to 17-AAG dramatically decreased the number and size of lipid droplets inside these cells (Figure 2A). This effect on cellular fat accumulation was not due to an increase of lipolysis given that 17-AAG treatment did affect neither basal nor isoproterenol induced lipolysis (Figure 2B). The insulin-induced glucose uptake, another feature promoted during adipogenesis, was also inhibited in presence of 17-AAG (Figure 2C).

To confirm that inhibition of lipid accumulation by 17-AAG was due to the inhibition of adipocyte differentiation, we assessed the expression of different molecular markers of adipogenesis in 3T3-L1 cells. By quantitative RT-PCR, we found that 17-AAG significantly decreased the expression of PPARγ which is a key transcription factor in adipogenesis, as well as its downstream targets lipoprotein lipase (LPL) and GLUT4 (Figure 2D). The reduction of the other master regulator of adipogenesis CEBPα at the RNA and protein level was not significant (Figure 2D to F). The expression level of the direct target of geldanamycin Hsp90 did not change in presence of 17-AAG (Figure 2E).

### Geldanamycin analogues prevent the high fat diet-induced fat expansion

**Figure 3:**
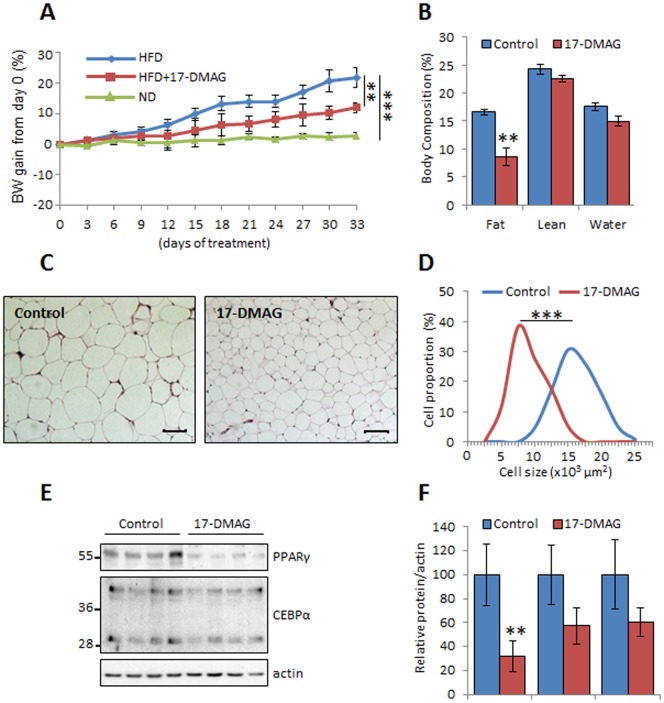
Geldanamycin analogues limit high-fat diet-induced adipose tissue expansion. (A to F) Two groups of mice HFD (n = 7) and HFD+17-DMAG (n = 8) were fed a HFD for 33 days and daily injected intraperitoneally with PBS or with 10 mg/kg of BW 17-DMAG. One group as control ND (n = 4) were fed with standard chow. (A) Percentage of body weight (BW) gain was measured. (B) Body composition of the mice fed with HFD or HFD+17-DMAG was evaluated by EchoMRI. (C) Histological analysis of the epididymal adipose tissue fixed and stained with hematoxylin and eosin. (D) Adipocytes cell surface was calculated using the ImageJ software, ***p<0.001. (E) White adipose tissue lysates were submitted to immunoblotting using antibodies against PPARγ, CEBPα and actin as a loading control. (F) The protein expression levels were quantified and values are given as mean ± SEM (n = 7 HFD, n = 8 HFD+17-DMAG), **p<0.01.

Given the antiadipogenic effects of geldanamycin analogues *in vitro* and the recently published observations on amelioration of obesity-induced renal failure by geldanamycin derivatives [24], we investigated whether geldanamycin analogues could modulate fat mass accumulation *in vivo*. C57BL6/N male mice were fed a HFD and treated with the water soluble 17-DMAG (10 mg/kg/day) to prevent side effects due to the solvent. After confirming that this selected dose had no effect on body weight in mice on normal chow diet, the geldanamycin analogue was injected intra-peritoneally daily for 33 days according to previously published pharmacokinetics data [25], [26]. We found that mice treated with 17-DMAG showed a significantly lower body weight (BW) gain compared to vehicle treated mice (Figure 3A, Figure S2). The injection of 17-DMAG has no significant effect on food intake, feces or urine production (Table 1), suggesting that at this selected dose, 17-DMAG did not cause anorexia, digestive or renal disorders. In addition, no major differences were observed concerning the locomotor activity, the behavior or depression between the 2 groups.

**Table 1:** Effects of GA treatment on physiological parameters of mice fed with HFD.

	NFD	HFD	HFD+17-DMAG	*p*
Food Intake (g/24 h)	3.66±0.33	3.80±0.10	4.00±0.09	0.508
Feces (g/24 h)	1.40±0.30	0.33±0.06	0.37±0.05	0.680
Urine/Water (24 h)	0.18±0.06	0.30±0.08	0.33±0.08	0.433
**Plasma**				
Glucose (mM)	10.83±0.85	13.47±0.83	10.88±0.60	0.056
Triglycerides (mM)	1.03±0.11	1.08±0.14	0.94±0.07	0.514
Cholesterol (mM)	4.06±0.22	4.17±0.17	3.96±0.13	0.159
**Urine**				
Na^+^ (mmol/24 h)	n.m	112.98±15.56	111.78±13.92	0.928
K^+^ (mmol/24 h)	n.m	267.79±35.06	296.78±31.36	0.623

Values are given as average ± SEM. The given *P* values for HFD vs. HFD+17-DMAG.

To examine whether the restricted BW gain was due to a limited fat mass accumulation, we evaluated the whole-body composition of the two groups of mice by EchoMRI. As shown in Figure 3B, the fat mass was significantly decreased (around 40%) in 17-DMAG treated mice, but not the lean mass or the total body water. Histological analysis of the epididymal adipose tissue revealed smaller and less heterogeneous adipocyte size in treated mice (Mean size 14830.3 μm^2^ SE = 282 vs. 8137.5 μm^2^ SE = 222) (Figure 3C and D). The same observations were done in the inguinal fat pad (Figure S3). Blood samples analysis revealed also a tendency towards reduction of glucose, triglycerides and cholesterol with values comparable to mice fed a normal fat diet (Table 1).

In accordance with 17-AAG effects *in vitro*, the expression levels of the master adipogenic transcriptional factors revealed a dramatic reduction of PPARγ and to lower extent CEBPα in fat tissue of mice treated with Hsp90 blocker (Figure 3E and 3F).

### Hsp90 blockers affect MR and GR expression in vitro and in vivo

**Figure 4:**
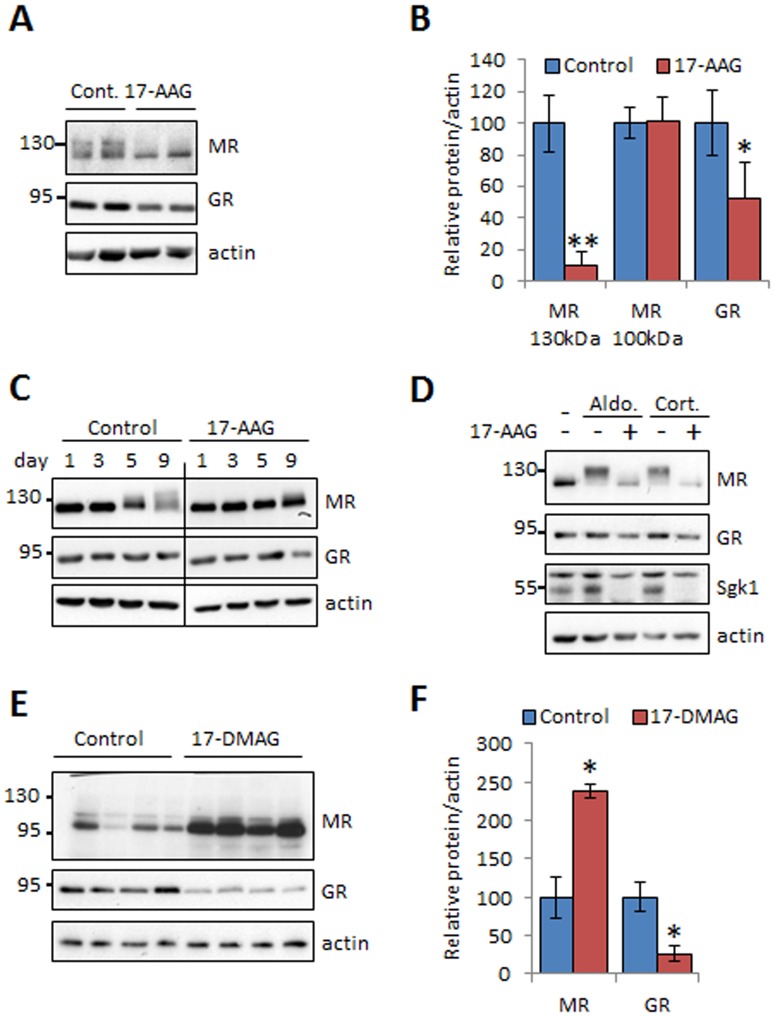
Geldanamycin analogues affect glucocorticoid receptors expression. (A to C) 3T3-L1 preadipocytes were induced to differentiation with or without 17-AAG (100 nM) for 10 days. (A and B) Cell lysates were analyzed by immunoblotting using antibodies against MR, GR and actin asa loading control. (B) The MR and GR expression level was quantified and values were normalized to actin and displayed as mean ± SD (n = 3), *p<0.05, **p<0.01. (C) 3T3-L1 preadipocytes were induced to differentiation and lysed at indicated day with or without 17-AAG treatment (100 nM) for 24 h. (D) Preadipocytes were treated with aldosterone (10 nM) or corticosterone (10 nM) with or without 17-AAG (100 nM) for 3 h before lysis. Cell lysates were then analyzed by immunoblotting using antibodies against MR, GR, and actin as a loading control. (E) White adipose tissue lysates from HFD or HFD+17-DMAG mice were submitted to immunoblotting using antibodies against MR, GR and actin as a loading control. (F) The protein expression levels were quantified and values are given as mean ± SEM (n = 7 HFD, n = 8 HFD+17-DMAG), *p<0.05.

It is known that geldanamycin analogues display a potent inhibitory effect on MR and GR activity [6], [7], [8]. To evaluate the effects of geldanamycin analogues treatment on these key Hsp90-bound receptors involved in adipogenesis, we first determined the expression level of MR and GR levels in mature 3T3-L1 adipocytes treated or not with 17-AAG from the beginning of the differentiation process. 17-AAG treatment decreased the expression of GR (Figure 4A and 4B), as it was described previously in other cell types [7]. Regarding MR, the expression profile diverged between the two conditions. In untreated cells, MR appeared as a doublet on immunoblot and 17-AAG treatment caused the disappearance of the upper band without major effects on the expression of the lower band (Figure 4A and 4B). The same observation was made with the specific MR blocker spironolactone (Figure S4A). However, higher doses of 17-AAG induced also a decrease in MR expression level (Figure S4B). Then we performed a time-course experiment to follow the expression pattern of MR during the differentiation process. As shown in Figure 4C, the upward shift of MR was clearly visible at day 9 after confluency and this modification was prevented by 24 h of 17-AAG treatment. We have recently demonstrated that the upper band represents the phosphorylated activated form of MR, which is induced after ligand binding [22]. To confirm this observation, we treated preadipocytes with MR ligands 10 nM aldosterone or 10 nM corticosterone in the presence or absence of 17-AAG. Steroid stimulations induced the phosphorylation of MR, as evidenced by an upward shift that was completely abolished by 17-AAG treatment (Figure 4D). Given that the final maturation steps of adipocyte differentiation were done in absence of hormones, our results suggested an autocrine steroids stimulation that could endogenously activate MR. This observation confirmed a recent study showing the existence of an autocrine steroids production in mature adipocytes [27].

To verify these observations *in vivo*, we evaluated the expression levels of MR and GR in fat tissue of mice treated or not with 17-DMAG. The treated group presented a marked reduction of GR expression of about 80% in adipose tissue compared to the control (Figure 4E and 4F). Of interest, the adipose expression of MR increased in mice treated with 17-DMAG conversely to what was observed in 3T3-L1 and 3T3-F442A adipocytes *in vitro* (Figure 4E and 4F).

### Geldanamycin analogues inhibit MR, GR and PPARγ transcriptional activity

**Figure 5:**
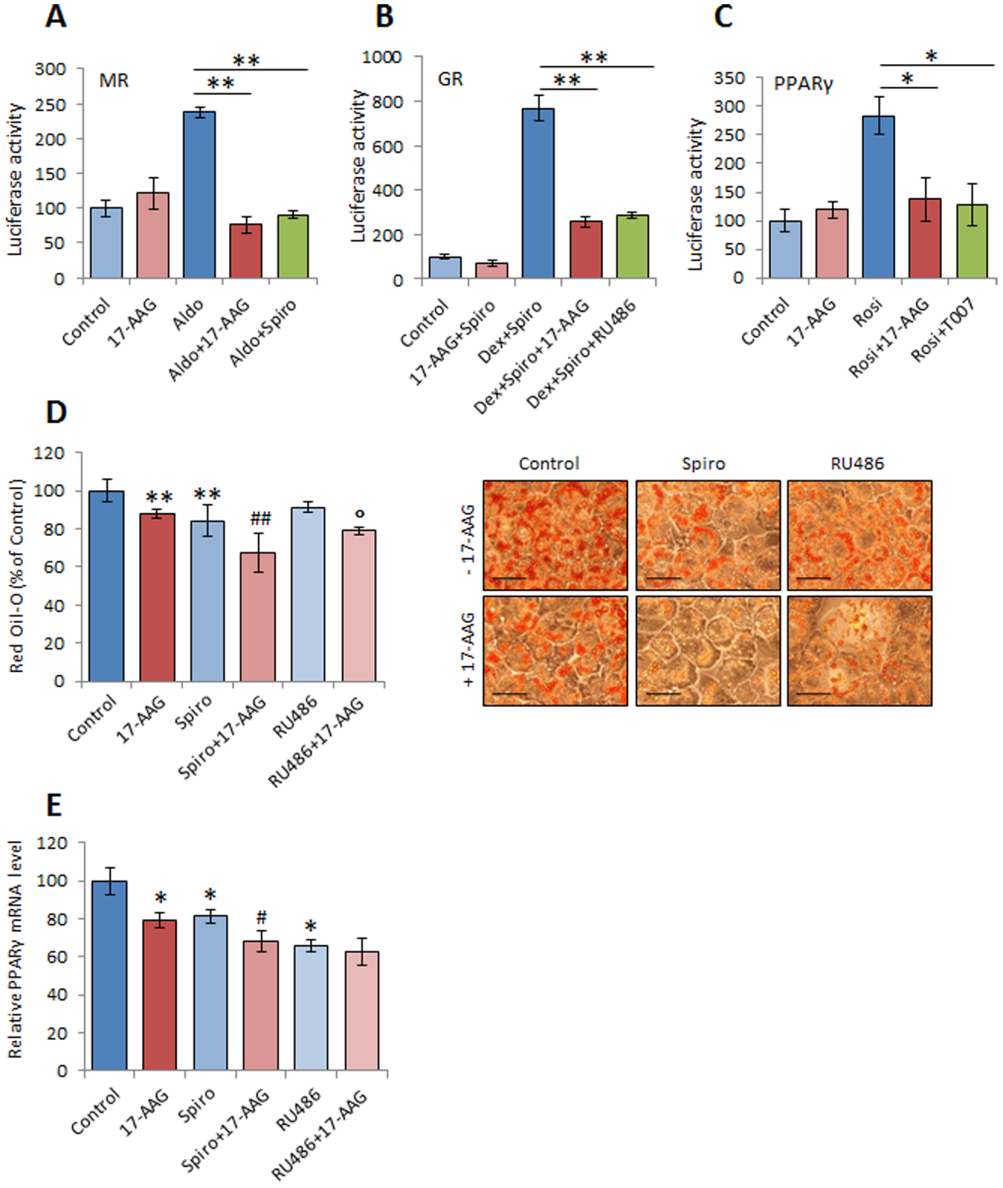
Hsp90 blockers inhibitMR, GR and PPARγ transcriptional activity in adipocytes. (A to C) Mature 3T3-L1 cells were co-transfected by Metafectene with the reporter genes MMTV-luc (for A and B), PRE-luc (for C), and the constitutive luciferase vector coding for Renilla. After 30 h, cells were exposed to the indicated treatment: 17-AAG (100 nM), aldosterone (100 nM), spironolactone (1 μM), dexamethasone (100 nM), RU486 (1 μM), Rosiglitazone (1 μM), T0070907 (3 μM) for 16 h. After hormone treatment, cells were washed with PBS and lysed with 200 μl of passive lysis buffer (Promega). Luciferase activity was determined by using a luciferase kit (Promega). Luciferase activity was normalized for constitutive Renilla luciferase. Values are given as mean ± SD (n = 3), *p<0.05, **p<0.01. (D and E) 3T3-L1 preadipocytes were induced to differentiate in absence or presence of spironolactone (10^−6^ M) or RU486 (10^−6^ M) alone or in combination with 17-AAG (100 nM) only for the first 3 days of differentiation protocol. (D) Red oil staining (scale bar, 70 μm) and analysis of triglyceride content.(E) PPARγ mRNA levels in 3T3-L1 preadipocytes after the first 3 days of differentiation.*p<0.05, **p<0.01 vs Control, ^#^p<0.05, ^##^p<0.01 vs Spiro, °p<0.05 vs RU486.

We have seen that reduction of PPARγ expression by geldanamycin analogues leads to a reduction in the expression of endogenous target genes like Glut4 or LPL (Figure 2C). The inhibition of MR phosphorylation and the decrease in GR expression level also abolished the steroidal induction of early target genes such as Sgk1 (Figure 4D). To confirm the effects of Hsp90 blockers on the transcriptional activity of MR, GR and PPARγ, we performed a luciferase reporter gene assay on mature 3T3-L1 cells. We found that the induction of the MMTV-luc reporter gene by 100 nM aldosterone was completely abolished by 17-AAG at similar extent as the MR inhibitor spironolactone (Figure 5A). To evaluate the specific GR activity, we used the same luciferase reporter gene and we co-stimulated the cells with 100 nM dexamethasone (that binds to GR and MR) and 1 μM spironolactone to suppress MR activity. We found that GR activation was also repressed by 17-AAG in a similar manner as the specific GR blocker RU486 (Figure 5B). Finally, to assess PPARγ activity, we found that 17-AAG as the PPARγ specific blocker T0070907 prevented the PPRE-luc reporter gene induction by 1 μM Rosiglitazone (Figure 5C).

It has been previously described that early exposure to aldosterone and dexamethasone enhance adipocyte differentiation and promote PPARγ expression [5], [10]. Therefore, we assessed the effect of Hsp90 blockers on the aldosterone and dexamethasone induced PPARγ expression. We found that treatment with 17-AAG from the beginning of the differentiation process, significantly inhibited the steroidal induction of PPARγ mRNA expression (Figure S5). Then, to evaluate the respective role of MR and GR as well as the importance of 17-AAG inhibition in the early steps of adipogenesis, we treated 3T3-L1 cells with spironolactone (1 μM) or RU486 (1 μM) alone or in combination with 17-AAG (100 nM) for the first 3 days of the differentiation protocol. We found that spironolactone treatment, but not RU486, significantly reduced lipid accumulation at day 9. Early exposure of cells to 17-AAG also reduced lipid accumulation, and such effect was further enhanced by spironolactone (Figure 5D).

To corroborate our findings at molecular level, we studied the effect of early 17-AAG, spironolactone and RU486 treatment on PPARγ expression. As shown in Figure 5E, all these compounds decreased PPARγ mRNA level; however, the inhibitory effect of 17-AAG was enhanced only by spironolactone. These results suggest that in addition to block the activity of PPARγ, geldanamycin analogues prevent its early *de novo* synthesis mediated by MR and/or GR.

### Inhibition of Hsp90 activity affects the production of adipokines

**Figure 6:**
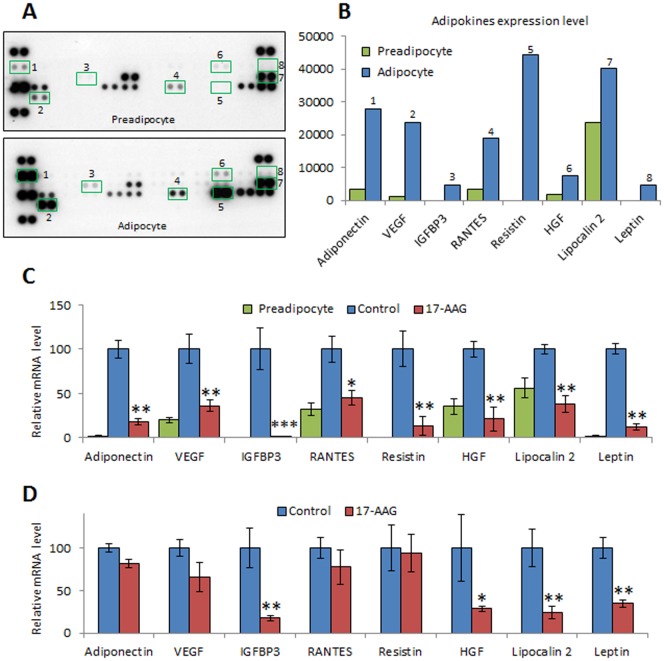
17-AAG affects adipokines production. (A) 3T3-L1 preadipocytes were induced or not to differentiation. 24 h supernatants from preadipocytes and mature adipocytes were collected and assessed for adipokines expression using an antibody adipokine array. The cytokine level was determined by chemiluminescence detection and autoradiography. Among the 38 cytokines tested, 8 were up-regulated in mature adipocytes. (B) Each dot of the array was quantified using Image J software. (C) 3T3-L1 preadipocytes were induced to differentiation in presence or absence of 17-AAG (100 nM) from the beginning of the differentiation process. The abundance of different adipokines mRNA was measured by quantitative RT-PCR. (D) 3T3-L1 mature adipocytes were treated or not with 17-AAG (100 nM) for 48 h. The abundance of different adipokines mRNA was measured by quantitative RT-PCR. Given are means (n = 3) relative to GAPDH ± SD, *p<0.05, **p<0.01, ***p<0.001 compared to non-treated mature adipocytes.

It is well documented that the secretion profile of adipocytes varies depending on their differentiation state or hormonal stimulation [28]. To determine the effect of Hsp90 inhibition on adipokines production, we first identified the differentially secreted adipokines in the cell culture supernatants from 3T3-L1 pre-adipocytes or mature adipocytes. Using an adipokine array, we found 8 adipokines that were up-regulated in mature adipocyte cells compared to preadipocytes (Figure 6A and 6B). Then we evaluated the expression of these identified adipokines in 3T3-L1 cells treated or not with 17-AAG from the beginning of the differentiation process. As shown in Figure 6C, 17-AAG treated cells presented a significant reduced expression of all the tested adipokines and displayed an adipokine profile close to the non-differentiated cells. To test whether GA could affect the adipokine profile in mature adipocytes, we treated fully mature adipocytes (day 10 after confluence) with 17-AAG for 48 hours. We found that treated adipocytes presented a different profile compared to control, with an significant reduction of IGFBP3, HGF, Lipocalin 2 and Leptin, but only a minor effect on Adiponectin, VEGF, Rantes and Resistin (Figure 6D).

## Discussion

Hsp90 blockers are frequently used to limit the function and expression of steroid and nuclear receptors [6], [7], [8], [29], [30]. Given the determinant role of nuclear receptors in adipogenesis, we investigated the effects of two geldanamycin analogues, the DMSO soluble 17-AAG and the water soluble 17-DMAG on adipogenesis. In this report, we showed that geldanamycin analogues prevented adipocyte differentiation by inhibiting the adipogenic transcriptional program that led to the reduction of lipid accumulation into the cell. These results are in line with very recent observations showing an inhibitory effect of Hsp90 blockers on 3T3-L1 differentiation [20], [21]. These findings were strengthened by our *in vivo* experiment showing a limited BW gain and fat mass expansion of 17-DMAG treated mice fed a HFD.

Geldanamycin is an inhibitor of the chaperone protein Hsp90. It binds with high affinity to the ATP binding pocket of Hsp90 and blocks the protein in its ADP-bound inactive status [31]. Hsp90 plays a crucial role in the ligand activation of steroid signaling pathways such as the aldosterone and glucocorticoid signaling. We and others have previously shown that geldanamycin analogues prevent the hormonal-induced activation of nuclear receptors such as MR and also GR by inducing their degradation via the ubiquitin-proteasome system [6], [7], [8]. If PPARγ is considered as the master regulator of adipogenesis, both MR and GR are also needed in the differentiation of adipocytes. However, their respective roles in the process of adipogenesis remain unclear. Whereas GR knock-down does not seem to have profound impact on adipocyte conversion, MR knock-down or MR blockers completely abolished lipid accumulation [10], [32]. Furthermore, unlike to GR, MR expression is induced during the preadipocyte differentiation and obese mice models (*ob/ob* and *db/db*) display a higher MR expression level compared to lean mice [14], [33]. In this study, we reported that activity of MR, and at a lower extent GR, in early adipogenesis are crucial for a full development of the maturation process of 3T3-L1 cells. We also demonstrated that the selected dose of Hsp90 blocker (100 nM) dramatically reduced PPARγ and GR expression levels and inhibited the phosphorylation of MR. These expression levels changes in GR and PPARγ, but not MR, were confirmed in mice treated with 17-DMAG. Nevertheless, the reporter gene assay allowed us to correlate the effects of geldanamycin analogues on the expression of these nuclear receptors with an inhibition of their transcriptional activity.

In our preliminary animal study, treatment with 10 mg/kg/day of 17-DMAG limited fat accumulation in mice fed a high fat diet without any relevant side effects. Our findings are corroborated by a recent study demonstrating that treatment with a lower dose of geldanamycin (6.5 μg/kg/day) preserved kidney function of obese mice fed a high fat diet [24]. Taken together, these observations allow us to suggest that Hsp90 blockers not only inhibit adipogenesis, but also prevent the negative effects of excessive fat mass accumulation on kidney function. This could be explained by the effects of geldanamycin analogues on the endocrine function of adipose tissue. Excessive growth of adipose tissue leads to disturbances of the adipokine profile, and this disregulation plays a central role in the appearance of the obesity-related metabolic disorders [34]. Here, we demonstrated *in vitro* that 17-AAG modified the production of adipokines in a different manner if it was administrated at the beginning of the differentiation process or later during adipogenesis. In both conditions, we found that Lipocalin 2, a direct target gene of MR, was down-regulated. Lipocalin 2 is a biomarker for human acute kidney injury and it has been shown to play an essential role for chronic kidney disease progression in mice and humans [35], [36].

Following our *in vitro* and *in vivo* experiments, the question arises whether geldanamycin analogues effects occurred via a direct MR and GR signaling down-regulation or via the disturbance of other nuclear receptors bound to Hsp90. Nguyen *et al*. proposed that PPARγ protein destabilization is the main mechanism by which Hsp90 inhibitors block the adipogenesis process [21]. However, we have found that geldanamycin analogues could also interfere with the induction of PPARγ mRNA during adipocyte differentiation as well as its induction by steroids. These observations suggest that in addition to decrease the available pool of PPARγ protein, inhibition of Hsp90 also prevents the *de novo*-synthesis of this transcription factor by inhibiting upstream signaling pathways. The Androgen Receptor (AR) represents another nuclear receptor that may be affected by Hsp90 inhibition. However, a potential effect through AR is excluded given that inhibition of AR signaling has no relevant effects on adipocyte differentiation *in vitro* [11], [37], but rather the deletion of AR gene *in vivo* increased susceptibility to visceral obesity [38]. Further investigations will lead to definitively elucidate the main cause of the anti-adipogenic effect of Hsp90 inhibitors. Given the numerous partners of this chaperone protein, it seems clear that its inhibition leads to the disturbance of the adipogenesis process at several steps. Furthermore, a full characterization and large metabolic studies in suitable animal models are required to confirm the efficiency of Hsp90 blockers on excessive fat mass accumulation and related metabolic disorders and the implication of glucocorticoids signaling in these effects.

In summary, our study demonstrated a powerful effect of geldanamycin analogues consisting in the inhibition of adipocyte differentiation and function *in vitro* and fat mass accumulation *in vivo*. These effects were paralleled with an inhibition of MR, GR and PPARγ signaling in adipocytes. Our study adds additional evidences and underlines the importance of glucocorticoids signaling inhibition for the prevention of obesity and its related metabolic complications.

## Supporting Information

Figure S1 **Geldanamycin Analogues inhibit lipid accumulation in adipocytes.** (A) At confluence, 3T3-L1 or 3T3-F442A preadipocytes were induced to differentiation in presence of an increasing dose of 17-AAG or 17-DMAG for 10 days. Lipid accumulation was visualized under microscope after Oil-Red-O staining. (B) The lipid accumulation was quantified after extraction of the stained lipid and the absorbance measured at 520 nm. Data are given as mean ± SD (n = 3), *p<0.05, ***p<0.001.
(PDF)

Figure S2 **17-DMAG limits high fat diet-induced body weight gain.** Two groups of mice HFD (n = 7) and HFD+17-DMAG (n = 8) were fed a HFD for 33 days and daily injected intraperitoneally with PBS or with 10 mg/kg of BW 17-DMAG. One group as control ND (n = 4) were fed a standard chow. The body weight was measured every 3 days.
(PDF)

Figure S3 **17-DMAG prevents adipocyte hypertrophy.** Histological analysis of the inguinal adipose tissue fixed and stained with hematoxylin and eosin. Visualized under light microscope (X10). Scale bar = 200 μm.
(PDF)

Figure S4 **Effects of blockers on adipocyte MR expression.** (A) 3T3-L1 preadipocytes were induced to differentiation with or without spironolactone (10^−5^ M) for 10 days. Cell lysates were analyzed by immunoblotting using antibodies against MR and actin as a loading control. (B) 3T3-L1 preadipocytes were induced to differentiation. At day 2 cells were treated with an increasing dose of 17-AAG for 24 h. Cell lysates were analyzed by immunoblotting using antibodies against MR, GR and actin as a loading control.
(PDF)

Figure S5 **17-AAG prevents steroid induction of PPARγ.** 3T3-L1 preadipocytes were induced to differentiation in presence or absence of 17-AAG (100 nM), aldosterone (10 nM) or dexamethasone (100 nM) for 10 days. The abundance of PPARγ mRNA was measured by quantitative RT-PCR. Given are means relative to GAPDH of 2 experiments performed in triplicate ± SD, **p<0.01.
(PDF)

Table S1 **Primer sequences used in the quantitative PCR experiments.**
(PDF)
