# Supplementary material for: Hsp90 Blockers Inhibit Adipocyte Differentiation and Fat Mass Accumulation
Source: PLoS One. 2014 Apr 4;9(4):e94127. doi: 10.1371/journal.pone.0094127 (PMC3976389; doi:10.1371/journal.pone.0094127)
Supplement: Figure S1 — Geldanamycin Analogues inhibit lipid accumulation in adipocytes. (A) At confluence, 3T3-L1 or 3T3-F442A preadipocytes were induced to differentiation in presence of an increasing dose of 17-AAG or 17-DMAG for 10 days. Lipid accumulation was visualized under microscope after Oil-Red-O staining. (B) The lipid accumulation was quantified after extraction of the stained lipid and the absorbance measured at 520 nm. Data are given as mean ± SD (n = 3), *p<0.05, ***p<0.001. (PDF) [file pone.0094127.s001.pdf]

Figure S1

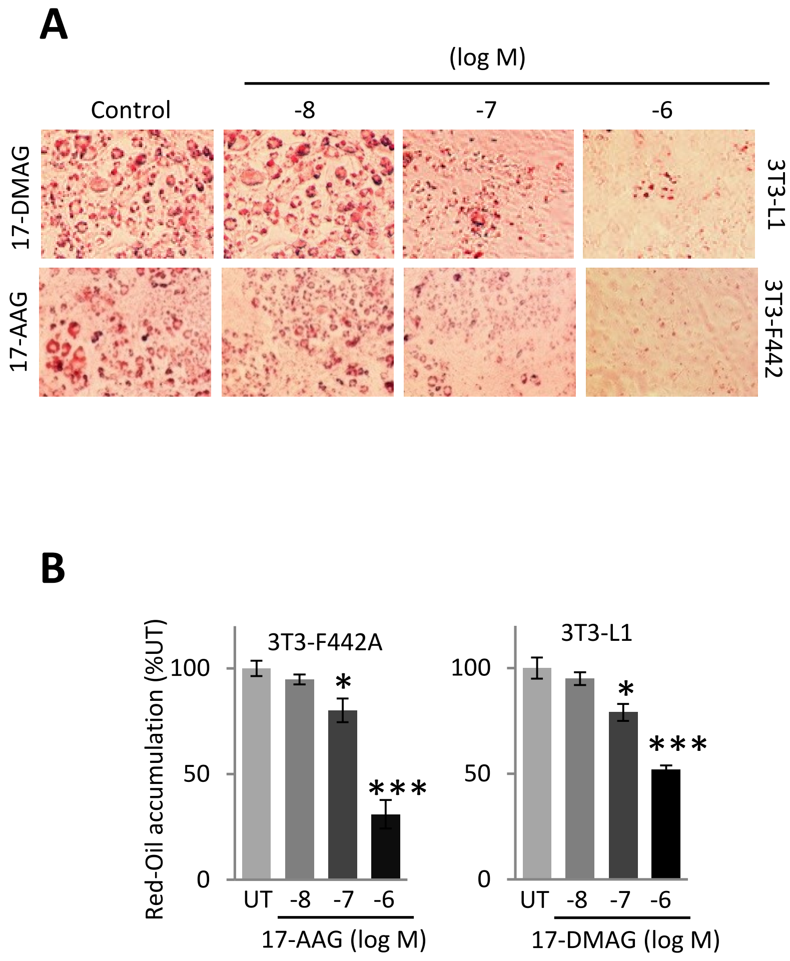

Geldanamycin Analogues inhibit lipid accumulation in adipocytes. (A) At confluence, 3T3-L1 or 3T3-F442A preadipocytes were induced to differentiation in presence of an increasing dose of 17-AAG or 17-DMAG for 10 days. Lipid accumulation was visualized under microscope after Oil-Red-O staining. (B) The lipid accumulation was quantified after extraction of the stained lipid and the absorbance measured at 520nm. Data are given as mean ± SD (n=3), *p<0.05, ***p<0.001
