# Supplementary material for: Hsp90 Blockers Inhibit Adipocyte Differentiation and Fat Mass Accumulation
Source: PLoS One. 2014 Apr 4;9(4):e94127. doi: 10.1371/journal.pone.0094127 (PMC3976389; doi:10.1371/journal.pone.0094127)
Supplement: Figure S2 — 17-DMAG limits high fat diet-induced body weight gain. Two groups of mice HFD (n = 7) and HFD+17-DMAG (n = 8) were fed a HFD for 33 days and daily injected intraperitoneally with PBS or with 10 mg/kg of BW 17-DMAG. One group as control ND (n = 4) were fed a standard chow. The body weight was measured every 3 days. (PDF) [file pone.0094127.s002.pdf]

Figure S2

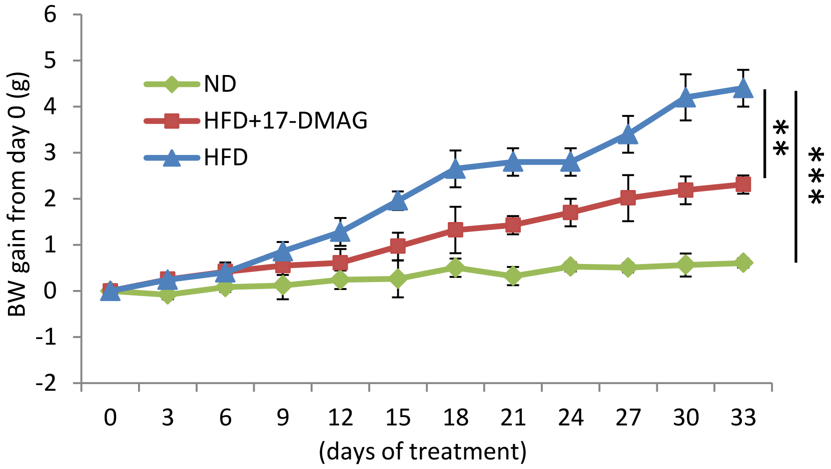

17-DMAG limits high fat diet-induced body weight gain. Two groups of mice HFD (n=7) and HFD+17-DMAG (n=8) were fed a HFD for 33 days and daily injected intraperitoneally with PBS or with 10mg/kg of BW 17-DMAG. One group as control ND (n=4) were fed a standard chow. The body weight was measured every 3 days.
