# Supplementary material for: Hsp90 Blockers Inhibit Adipocyte Differentiation and Fat Mass Accumulation
Source: PLoS One. 2014 Apr 4;9(4):e94127. doi: 10.1371/journal.pone.0094127 (PMC3976389; doi:10.1371/journal.pone.0094127)
Supplement: Figure S3 — 17-DMAG prevents adipocyte hypertrophy. Histological analysis of the inguinal adipose tissue fixed and stained with hematoxylin and eosin. Visualized under light microscope (X10). Scale bar = 200 μm. (PDF) [file pone.0094127.s003.pdf]

Figure S3

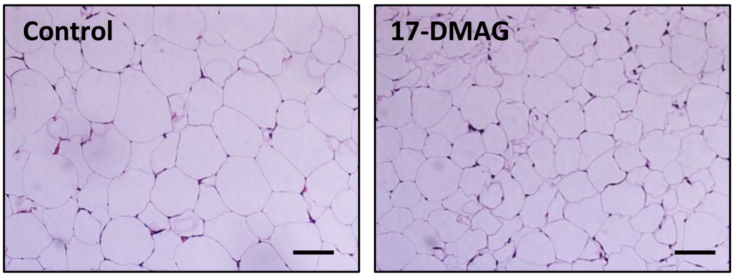

17-DMAG prevents adipocyte hypertrophy. Histological analysis of the inguinal adipose tissue fixed and stained with hematoxylin and eosin. Visualized under light microscope (X10). Scale bar = 200µm
