# Supplementary material for: Hsp90 Blockers Inhibit Adipocyte Differentiation and Fat Mass Accumulation
Source: PLoS One. 2014 Apr 4;9(4):e94127. doi: 10.1371/journal.pone.0094127 (PMC3976389; doi:10.1371/journal.pone.0094127)
Supplement: Figure S4 — Effects of blockers on adipocyte MR expression. (A) 3T3-L1 preadipocytes were induced to differentiation with or without spironolactone (10−5 M) for 10 days. Cell lysates were analyzed by immunoblotting using antibodies against MR and actin as a loading control. (B) 3T3-L1 preadipocytes were induced to differentiation. At day 2 cells were treated with an increasing dose of 17-AAG for 24 h. Cell lysates were analyzed by immunoblotting using antibodies against MR, GR and actin as a loading control. (PDF) [file pone.0094127.s004.pdf]

Figure S4

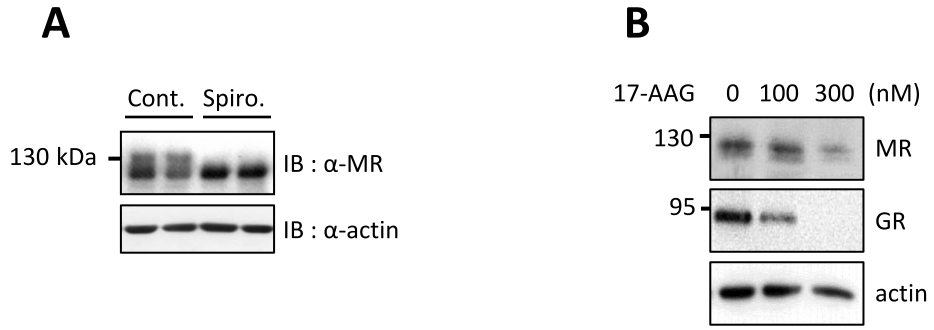

Effects of blockers on adipocyte MR expression. (A) 3T3-L1 preadipocytes were induced to differentiation with or without spironolactone (10^{-5} M) for 10 days. Cell lysates were analyzed by immunoblotting using antibodies against MR and actin as a loading control. (B) 3T3-L1 preadipocytes were induced to differentiation. At day 2 cells were treated with an increasing dose of 17-AAG for 24h. Cell lysates were analyzed by immunoblotting using antibodies against MR, GR and actin as a loading control.
