# Supplementary material for: Hsp90 Blockers Inhibit Adipocyte Differentiation and Fat Mass Accumulation
Source: PLoS One. 2014 Apr 4;9(4):e94127. doi: 10.1371/journal.pone.0094127 (PMC3976389; doi:10.1371/journal.pone.0094127)
Supplement: Figure S5 — 17-AAG prevents steroid induction of PPARγ. 3T3-L1 preadipocytes were induced to differentiation in presence or absence of 17-AAG (100 nM), aldosterone (10 nM) or dexamethasone (100 nM) for 10 days. The abundance of PPARγ mRNA was measured by quantitative RT-PCR. Given are means relative to GAPDH of 2 experiments performed in triplicate ± SD, **p<0.01. (PDF) [file pone.0094127.s005.pdf]

Figure S5

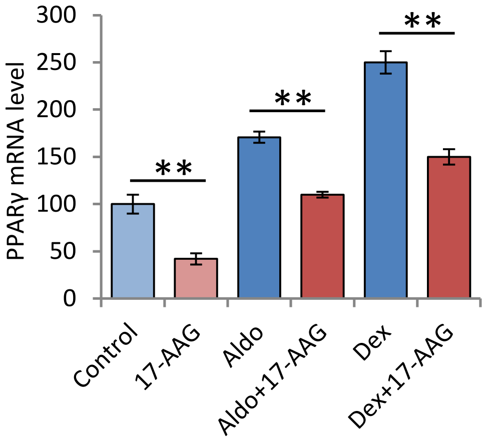

17-AAG prevents steroid induction of PPARγ. 3T3-L1 preadipocytes were induced to differentiation in presence or absence of 17-AAG (100 nM), aldosterone (10 nM) or dexamethasone (100 nM) for 10 days. The abundance of PPARγ mRNA was measured by quantitative RT-PCR. Given are means relative to GAPDH of 2 experiments performed in triplicate ± SD, **$p<0.01$.
