# Supplementary material for: Hsp90 Blockers Inhibit Adipocyte Differentiation and Fat Mass Accumulation
Source: PLoS One. 2014 Apr 4;9(4):e94127. doi: 10.1371/journal.pone.0094127 (PMC3976389; doi:10.1371/journal.pone.0094127)
Supplement: Table S1 — Primer sequences used in the quantitative PCR experiments. (PDF) [file pone.0094127.s006.pdf]

Table S1

Primers	Sequences
PPARγ fwd	TTC ACA AGA GCT GAC CCA AT
PPARγ rev	AAG CCT GAT GCT TTA TCC CC
CEBPα fwd	GAC AAG AAC AGC AAC GAG TA
CEBPα rev	AGC TGG CGG AAG ATG C
Glut4 fwd	ACACTGGTCCTAGCTGTATTCT
Glut4 rev	CCAGCCACGTTGCATTGTA
LPL fwd	GCTCTCAGATGCCCTACAAA
LPL rev	GATGTCCACCTCCGTGTAAA
Adiponectin fwd	TGC CGA AGA TGA CGT TAC TA
Adiponectin rev	TCT CAC CCT TAG GAC CAA GA
VEGF fwd	GTG GAC ATC TTC CAG GAG TAC C
VEGF rev	TGC TGT AGG AAG CTC ATT CTC T
IGFBP3 fwd	CAC GGA GCT GGT GCG
IGFBP3 rev	TGC TTA GGC TGC CTG C
Rantes fwd	CCT CAC CAT CAT CCT CAC TG
Rantes rev	CAC ACA CTT GGC GGT TC
Resistin fwd	AAC AAG ACT TCA ACT CCC TG
Resistin rev	TGT CCA GTC TAT CCT TGC AC
HGF fwd	GGT GTT TCA CAA GCA ATC CA
HGF rev	GCC CTT GTC GGG ATA TCT TT
Lipocalin 2 fwd	GTC GCT ACT GGA TCA GAA CA
Lipocalin 2 rev	CTT GGT TCT TCC ATA CAG GGT
Leptin fwd	CTG GCA GTC TAT CAA CAG GTC
Leptin rev	TCC ACC TCT GTG GAG TAG AG

Primer sequences used in the quantitative PCR experiments.
